# Assessing effectiveness of a novel mid-upper arm circumference Z-score tape in a community setting in Guatemala

**DOI:** 10.1186/s13690-019-0370-0

**Published:** 2019-10-04

**Authors:** Mikaela A. Miller, Kristen Mallory, Manolo Escobedo, Ana Cecilia Tarot, Susan Abdel-Rahman

**Affiliations:** 1 Children International, Kansas City, USA; 2 Children International, Guatemala City, Guatemala; 3 Children’s Mercy Hospital, Kansas City, USA

**Keywords:** Acute malnutrition, Mid-upper arm circumference, Community screening of malnutrition

## Abstract

**Background:**

Mid-Upper Arm Circumference (MUAC) is an independent anthropometric measurement used to identify malnutrition in children. While much research has been dedicated to applying fixed estimates of MUAC to identify cases of malnutrition in children under 5 years of age, far less has been done with age-specific MUAC Z-score values across the continuum of age from birth through adolescence.

**Methods:**

The present study examined the effectiveness of a novel MUAC Z-score tape, in the hands of community health volunteers, to identify children over the age of 5 who would benefit from nutritional rehabilitation. In January of 2019, 112 community health volunteers working within Children International in Guatemala were trained to use the MUAC Z-score tape and asked to collect measurements on children or youth in their communities.

**Results:**

Of the 818 MUAC Z-score tape measurements obtained by volunteers, 88.26% (722/818) were concordant with nutritional risk status as predicted by BMI Z-score, and 90.95% (744/818) were concordant with MUAC Z-score tape measurements made by field medical staff. MUAC Z-scores identified 87.10% (27/31) of the severely or moderately undernourished children as determined by the BMI Z-score who would be candidates for the nutrition rehabilitation program (Z-score ≤ − 2) along with an additional six children that would not have been classified as such with BMI Z-score. A qualitative survey distributed to the volunteers showed moderate rates of understanding of nutritional risk using the tape, and 62.50% reported the tape was easy to use.

**Conclusions:**

These quantitative and qualitative findings suggest that with more in-depth training and education the MUAC Z-score tape is a viable, low-cost, low-burden alternative for community-level nutritional status assessment among the population served by Children International in Guatemala.

**Supplementary information:**

**Supplementary information** accompanies this paper at (10.1186/s13690-019-0370-0).

## Background

Accurate and timely identification of children suffering from, or at risk of, severe acute malnutrition (SAM) is essential to direct them to appropriate care. The deleterious and potentially irreversible effects of undernutrition on growth and cognitive development have been well documented in children under the age of five, and an estimated 45% of mortality in this age range is attributable to undernutrition [1–4]. Although most intervention work focuses on the critical window from conception to 24 months, an expanding body of evidence suggests that growth catch-up can occur during middle and late childhood and adolescence [5]. This period of life is marked by significant physical, neurological, and social development. Executive neurological function and frontal lobe development is thought to occur in late childhood and adolescence, and adequate nutrient intake is highly associated with cognitive performance and maintenance, as well as educational achievement [6–8]. Physiologically, underweight school-aged children are more likely to suffer from delayed onset of puberty, reduced bone density, deficient muscular development, and poorer overall health which may impact their ability to work later in life [9]. The effects of malnutrition persisting into adolescence and the reproductive years can extend to subsequent generations, adversely affecting fetal development and the epigenome of their children [10]. Thus, undernutrition in school-aged children and adolescents exerts considerable strain on the health, education, and economic systems in low and middle income countries [11].

Undernutrition is most prevalent in low-resource settings where unskilled staff, inadequate tools, and insufficient time hinder nutritional screening program success. Detection methods should, therefore, be simple for delivery at the community level [12]. Various anthropometric measurements have been utilized to identify high risk cases, of which Mid-Upper Arm Circumference (MUAC), Weight-for-Height (WH) Z-scores in children under 2, and Body Mass Index (BMI) Z-score for ages 2–19 are the most widely used and debated [13, 14]. MUAC, as measured in mm, is an easier method to deploy in a community setting. WH/BMI Z-scores are utilized more often in health care settings where the tools required for obtaining weight and height are accessible. The cutoffs using WH/BMI Z-scores are well-established: SAM is identified by a Z-score < − 3, and moderate acute malnutrition (MAM) is identified as a Z-score between − 2 and − 3 [15]. Cutoffs for SAM and MAM using traditional MUAC tapes are much less consistent. A systematic review in 2013 concluded that MUAC could be adequately used as a stand-alone criterion for hospital admission and discharge due to SAM, but the MUAC cutoffs ranged from 110 to 130 mm for children aged 6 to 59 months [16]. Furthermore, independent studies offer contradictory evidence as to which measurement is more predictive of mortality due to malnutrition. Chiabi et al. concluded that MUAC was more predictive of mortality in children with SAM than WH Z-score [17], whereas Grellety and Golden recently determined that current MUAC methods based on mm cutoffs for children under the age of 5 inadequately identified individuals with the highest risk of mortality based on WH [18]. Briend et al. found that MUAC was preferable to WH but did not add predictive value [19]. The disparities may be attributable in part to the various cutoffs used by each group. Other studies have attempted to determine more appropriate cutoffs in various populations using an endpoint other than mortality, but these were based solely on the MUAC expressed in mm, and/or made for use in children ages 0–5 [20–29]. Accordingly, trade-off between ease of implementation in resource-limited settings, unacceptable rates of detrimental false negatives, and the burden of false positives needs to be addressed so that better protocol can be established and scaled.

To address limitations of traditional MUAC, several groups independently developed reference growth curves for age-specific MUAC Z-scores using the Health Examination Survey (HES) and the National Health and Nutrition Examination Survey (NHANES) [30–32]. Application to an East African population offered evidence that MUAC Z-score growth curves were at least as effective as BMI Z-score in 5–19 year-olds with or without comorbid HIV infection. Data from U.S. cohorts extended the MUAC Z-score to include children aged 2 months to 20 years, and provided the first indication that MUAC Z-score and BMI Z-score thresholds may require refinement [30, 33]. To facilitate screening, the Kansas City group also constructed MUAC Z-score tapes that use easily interpretable colors to indicate nutritional risk status. The MUAC Z-score tapes have been evaluated in practice by registered dietitians on a group of over 10,000 patients seen at their institution between October 2015 and October 2017 [33].

The MUAC Z-score tape has yet to be evaluated in the hands of health volunteers in a resource-limited community setting. Increasing accessibility of the MUAC tapes for community health workers and volunteers is an important step to increase program coverage, both in detecting SAM and treating uncomplicated SAM in a community setting [34]. Notably, there is some evidence to suggest that mothers are able to more accurately take and read traditional MUAC for their children than community health workers, which indicates household community monitoring may be as or more effective than community health worker screening [35]. The present study examined the effectiveness of the MUAC Z-score tape described above [32, 33] in the hands of non-medical volunteers to identify children over the age of 5 who would benefit from nutritional rehabilitation (Z–score < –2).

## Methods

### Device

Two versions of the MUAC Z-score tape were available for use, an infant tape spanning 2–59 months and one for children spanning 5–18 years. For this study we used the children’s tape with discrete age groups of 5, 5½, 6, 6½, 7, 7½, 8, 8½, 9, 9½, 10, 10½, 11, 12, 13, 14, 15, 16, 17, and 18 years (see Additional file 1). The device was constructed using flexible, tear-resistant paper and printed by Hallmark Cards, Inc. (Kansas City, MO). Down the center of the device is a traditional measuring scale depicting centimeters and millimeters. Above and below this scale are a series of color-coded bands demarcating the Z-score range into which the child’s MUAC falls for each age group. Markings appear on both sides and genders were pooled to mitigate the need for multiple versions of the device. After production, the tapes were checked for dimensional accuracy using a National Institute of Standards and Technology (NIST) certified ruler in compliance with ISO 9000 standards.

### Study design

Data were collected at the Tierra Nueva community center in the Guatemala agency of Children International (CI). CI is a mission-driven organization based in Kansas City, MO, USA that works in ten countries to end poverty through local partnerships, child and youth programming, and community involvement in the areas of health, education, empowerment, and employment. Tierra Nueva is one of seven community centers in the CI Guatemala agency and is located in Guatemala City. The population served by this community center is primarily urban and between 5 and 19 years of age.

CI health volunteers, primarily comprised of mothers and caregivers, were trained to use the MUAC Z-score tape at the Tierra Nueva community center. Training consisted of a 3-h session on the morning of January 11, 2019. Each volunteer received a MUAC Z-score tape and an instructional document that described how to use and interpret the tape. The Field Medical Officer presented general theory behind the tape and answered volunteers’ questions. The volunteers then practiced using the tape on each other before breaking into small groups to practice with children who were present at the community center. During this practical training portion those volunteers who were adept with the tape served as coaches for the others in the sector (approximately 15 volunteers per sector). Volunteers were trained on how to complete the tracking sheet, data collection protocols, and the plan for the experiment. Each volunteer was asked to complete measurements in children over the age of 5 years in their communities and instructed to record the data per the protocol over the next two weeks.

MUAC Z-score tape color and measurements in mm were collected on paper by the health volunteers, and the data entered into a spreadsheet template by the Field Health Coordinator. The same children were then invited to the community center to obtain current height, weight, and MUAC Z-score tape measurements within 15 days, to be completed by the field medical staff. All health volunteers were invited to complete a survey about their experiences using the MUAC Z-score tape (see Table 2).

### Statistical analysis

Standard descriptive statistics were used to summarize demographic and anthropometric characteristics. Continuous variables are reported with the median and interquartile range. Categorical variables are reported as percentage and counts represented by each level. BMI Z-scores were calculated for participants using the World Health Organization’s (WHO) Child Growth Standards macro in R [36]. BMI Z-scores were categorized into 7 risk categories based on standard deviation intervals derived from the WHO 2007 growth standards [15] (Table 1).

**Table 1:** Z-score risk category ranges for the study

Risk category	Z-score range
Severely malnourished	Z < -3
Moderately malnourished	-3 ≤ Z < -2
At risk of underweight	-2 ≤ Z < -1
Normal	-1 ≤ Z < 1
At risk of overweight	1 ≤ Z < 2
Overweight	2 ≤ Z < 3
Obese	Z ≥ 3

Agreement between pairs of anthropometric measurements concerning nutritional status (as predicted by BMI Z-score, MUAC Z-score range color measured by field medical staff, and MUAC Z-score range color measured by health volunteers) was assessed using Cohen’s Weighted Kappa for ordinal responses (quadratic weights). Adjusted bootstrap confidence intervals for Cohen’s Weighted Kappa were calculated using the BCa bootstrap method [37] with a *P*-value for Cohen’s Weighted Kappa of 0.05 or less indicating significant statistical agreement. McNemar’s Test of concordance (continuity corrected) was used to assess agreement between volunteer and BMI-predicted nutritional status in identifying nutrition rehabilitation candidates. Rejection of the null hypothesis for McNemar’s Test would provide sufficient evidence to conclude disagreement.

Characteristics of the child or health volunteer were examined using a generalized linear mixed model (link = logit) to determine if there was an association with misclassification and any of these factors. A random intercept was included for each health volunteer to account for intra-rater covariance. Polynomial terms for BMI Z-score were also examined up to degree 3. The normal approximation (Pr|Z|) was used to evaluate the significance of individual factors. An alpha-level of 0.05 was used to determine statistical significance for all tests. The statistical package R was used to conduct the analysis (version 3.5.2).

## Results

### Tierra Nueva quantitative results

In total, 112 volunteers were trained and 56 obtained MUAC Z-score tape measurements on 889 children and youth, of which 818 were able to complete follow-up height and weight measurements during the study duration. The remaining volunteers who did not contribute data to the study live in sectors with mostly older youth who would not have been considered candidates for the nutritional rehabilitation program. The median number of measurements completed by each community volunteer was 13.00 (IQR = 7.00–20.00). Child age ranged from 6 to 13 years (median = 10.75 years, IQR = 9.25–12.00). There were slightly more females (424/818, 51.83%) than males (394/818, 48.17%) that were measured with the MUAC Z-score tapes. Evidence of stunting was not as prevalent in the Tierra Nueva population (147/818, 17.97%), though Guatemala has one of the highest rates of stunting in the Western Hemisphere [38].

**Fig. 1:**
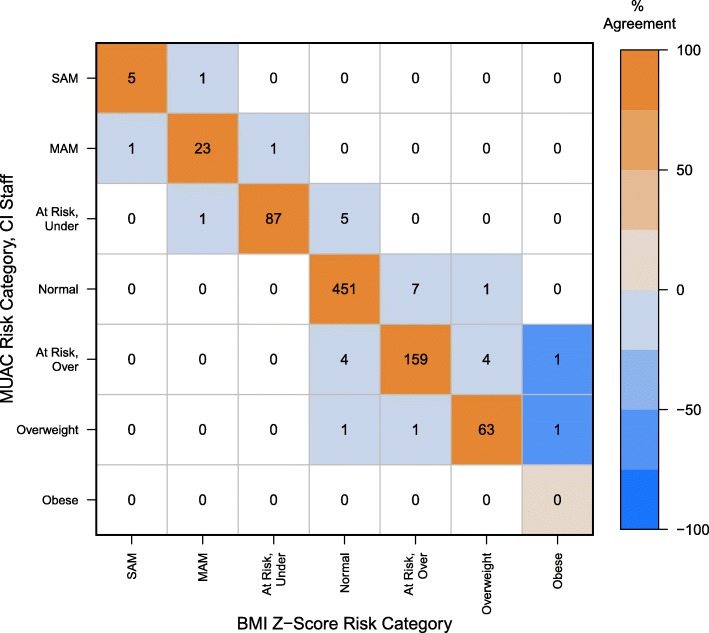
Predicted BMI Z-score and MUAC Z-score risk categories as measured by CI staff. The confusion matrix displays the counts in the cells, and color indicates agreement. Negative values on the off diagonals signify disagreement and vary in intensity of blue, whereas positive values on the diagonal represent agreement and vary in intensity of orange. For example, the cell at the intersection of BMI Z-score “Obese” and MUAC-CI Staff “At Risk, Over” is darker blue because there is 50% disagreement (1/2 measurements misclassified at that level). In contrast, BMI Z-score “Normal” and MUAC tape “At Risk, Over” is very light blue because the disagreement at that level is only 1% (4/461 misclassified at that level)

**Fig. 2:**
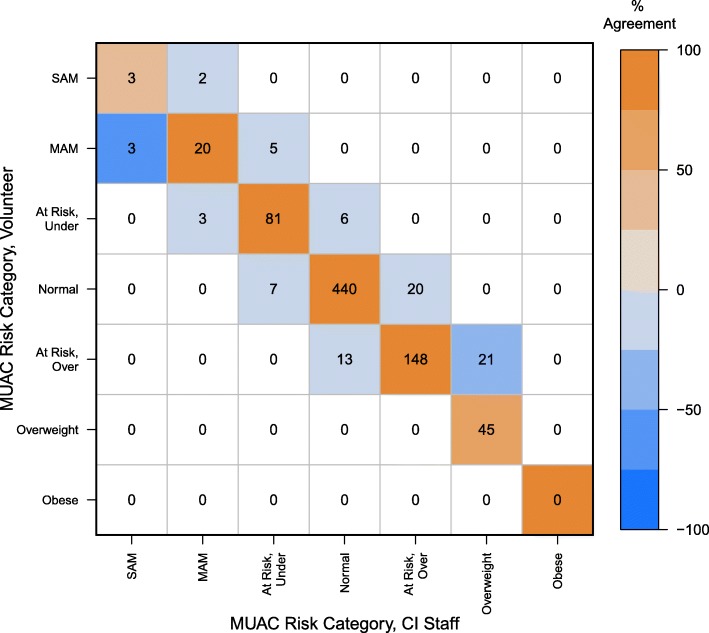
MUAC Z-score color risk category as measured by staff versus health volunteers. The confusion matrix displays the counts in the cells, and color indicates agreement. Negative values on the off diagonals signify disagreement and vary in intensity of blue, whereas positive values on the diagonal represent agreement and vary in intensity of orange. For example, the cell at the intersection of MUAC-CI Staff “SAM” and MUAC-Volunteer “MAM” is lighter orange because there is 50% disagreement (3/6 measurements misclassified at that level). In contrast, MUAC-CI Staff “Normal” and MUAC-Volunteer “At Risk, Over” is very light blue because the disagreement at that level is only 3% (13/459)

**Fig. 3:**
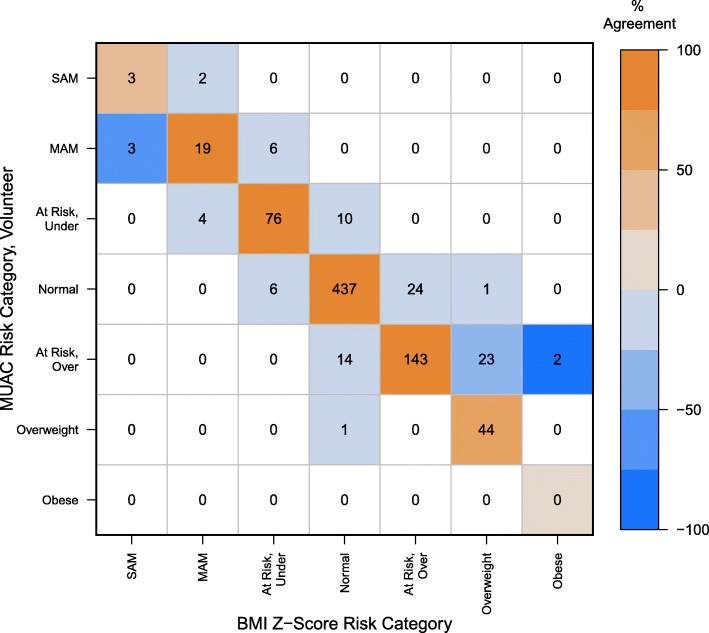
Predicted BMI Z-score and MUAC Z-score risk categories as measured by volunteers. The confusion matrix displays the counts in the cells, and color indicates agreement. Negative values on the off diagonals signify disagreement and vary in intensity of blue, whereas positive values on the diagonal represent agreement and vary in intensity of orange. For example, the cell at the intersection of BMI Z-score “SAM” and MUAC-Volunteer “MAM” is dark blue because there is 50% disagreement (3/6). In contrast, MUAC-Volunteer “Normal” and BMI Z-score “Overweight” is very light blue because the disagreement at that level is only 1% (1/68)

The highest level of concordance (96.33%, 788/818) was observed between the BMI Z-score predicted risk category and the MUAC Z-score category as measured by the field medical staff. The result was statistically significant with near-perfect agreement (Κ = 0.95; Z = 27.18, 95% CI (0.92, 0.97), *P* ≤ 0.0001) (Fig. 1). A concordance rate of 91.00% (744/818) was observed between MUAC Z-score tape measurements made by volunteers and those made by the field medical staff (Fig. 2), suggesting an exceptionally high level of statistically significant agreement (Κ = 0.88; Z = 25.26, 95% CI (0.85, 0.91), *P* ≤ 0.0001). We observed a concordance rate of 88.26% (722/818) with nutritional status predicted by MUAC Z-score as measured by the health volunteer versus BMI Z-score (Fig. 3). The results indicate a high level of agreement between nutritional status predicted by these two anthropometric measurements, albeit the lowest of the pairwise comparisons (Κ = 0.84; Z = 24.13, 95% CI (0.80, 0.87), *P* ≤ 0.0001).

Across all nutritional categories, we did not conclude significant disagreement between the nutritional status assigned by BMI Z-score and that assigned by MUAC Z-score tape (χ^2^(1) = 0.10, *P* = 0.75) with a concordance rate of 98.78% (808/818). In terms of correctly identifying candidates for the nutrition rehabilitation program, we concentrated on the agreement between BMI Z-score and MUAC Z-score for SAM and MAM as a combined category (Table 2). BMI Z-score assigned 31 children to MAM/SAM whereas MUAC Z-score assigned 33 children to MAM/SAM, with 27 children categorized by both measures. The individuals missed by the MUAC Z-score tape were all labeled as “at risk” of undernutrition according to predicted BMI Z-score, and two of the 4 were measured by the same volunteer. The MUAC Z-score tape identified an additional 6 individuals as potential candidates for the nutrition rehabilitation program that BMI z-score did not identify, all of which were labeled as “at risk” of undernutrition according to BMI z-score.

**Table 2:** Confusion matrix for nutritional rehabilitation program candidates

	MUAC Tape Color-Volunteer		
		Not a candidate	Candidate
BMI Z-score	Not a candidate	781	6
	Candidate	4	27

Candidates would fall in the two lowest categories (−2 SD on either scale, BMI Z-score or MUAC Z-score)

**Fig. 4:**
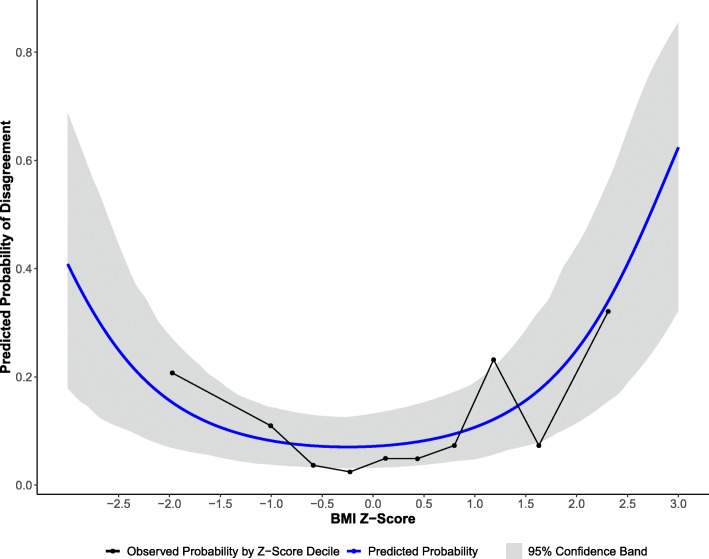
Model predicted versus observed probability of disagreement. The figure shows the model results between BMI Z-score and MUAC Z-score measured by volunteers (by BMI Z-score quantiles)

Model comparison tests determined that the model with a cubic term for BMI Z-score did not significantly improve model fit (χ^2^(1) = 0.85, *P* = 0.36), but the quadratic model was significantly better than the linear model (χ^2^(1) = 43.70, *P* < 0.0001). The results of the generalized linear mixed effect model revealed no significant association between age in months and odds of agreement between BMI Z-score and MUAC Z-score categories (OR: 1.05; 95% CI (0.84, 1.33); *P* = 0.65). Gender was also not a significant factor affecting the odds of agreement (OR: 0.92; 95% CI (0.58, 1.44); *P* = 0.70). While there was no evidence that height-for-age Z-score was associated with odds of agreement (OR: 0.87, 95% CI (0.68, 1.09), *P* = 0.23), BMI Z-score was significantly associated with agreement in a quadratic curvilinear manner (Fig. 4, *P* < 0.0001). On average, a one-unit increase in BMI Z-score (i.e., 1 standard deviation) resulted in 79.44% increase in the y-x slope in odds of agreement (OR: 1.79, 95% CI (1.52, 2.14)). Effectively, this means that probability of disagreement increases at the extremes of BMI Z-score.

### Tierra Nueva qualitative results

Surveys were completed by all 56 volunteers who contributed measurements to the study. Results of the survey questions appear in Table 3. When asked how difficult it was to measure the circumference of the arm with the MUAC Z-score tape, 62.50% (35/56) reported that it was either “easy” or “very easy.” A similar proportion (34/56) reported that it was either “easy” or “very easy” to understand nutritional risk status with the tape. Seventy-one percent of respondents (40/56) preferred the MUAC Z-score tape to the traditional method of height and weight with an additional 25.00% reporting that they did not have sufficient information with respect to other methods to make a decision (14/56). Respondents had the opportunity to provide other comments or suggestions for improvement. The most common comment about how to improve the tape was the suggestion to change in the material (24/56, 42.86%). Respondents also mentioned that the device should be available in the users’ native language, in our case Spanish (3/56, 5.36%).

**Table 3:**
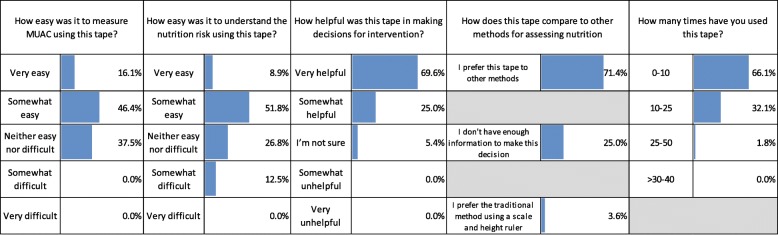
Survey results from the volunteers who contributed to the study

## Discussion

The quantitative and qualitative findings suggest that the paper-based MUAC Z-score tape is a viable, low-cost alternative for assessing nutritional status among the community served by Children International in Guatemala. The present method of identifying cases of moderate and severe acute malnutrition (BMI Z-score) places the onus on time-constrained medical personnel and other field staff with little or no medical experience. It also requires special equipment that must either be taken into rural communities or kept at a community center that is often many miles from the homes of beneficiaries. The single-step MUAC Z-score tape is much faster and easier to understand than collecting height and weight, calculating BMI, converting BMI to an age- and gender-relevant Z-score, and assigning nutritional risk based on that value.

These results further suggest that MUAC Z-score measurements completed by a corps of health volunteers is an effective way to screen for malnutrition. Volunteers with little to no medical experience were able to achieve 91.00% agreement with field medical staff, 88.26% agreement with BMI Z-scores, and 87.10% agreement in identifying underweight candidates for the nutrition rehabilitation program according to BMI Z-scores. However, only about half of the individuals with SAM were identified by both methods (Fig. 3, top left corner). While there are few observations in the extreme nutritional risk categories on either end, the model results provide evidence that probability of disagreement is more likely to occur the more extreme the measurement. This finding is consistent with a previous observation in U.S. children which concluded that Z-score thresholds may require refinement to improve concordance [33].

Importantly, this prior investigation also reported rates of concordance between both BMI Z-score and MUAC Z-score with clinical assessments of malnutrition raising questions of which measure is more sensitive for malnutrition [33]. There was no significant association between gender, age, or stunting (height-for-age Z-score < -2) with probability of disagreement. Only BMI Z-score (quadratic term) was found to be associated with probability of disagreement.

Our findings contribute to the current body of knowledge concerning the effectiveness of MUAC Z-score tape in identifying malnutrition in school-aged children and adolescents in the following ways. Firstly, this study represents one of the first instances of external validation of age-specific MUAC Z-scores using the novel tape in children aged 5 to 19 years in a community setting. Second, these results suggest that measurements completed by volunteers with little to no medical experience are in high agreement with those completed by the field medical staff and with the nutritional risk status as predicted BMI Z-scores. Third, a qualitative survey showed that the MUAC Z-score tape is easy to use and increases understanding of nutritional risk, although there is room for improvement in training and in the characteristics of the tape itself.

Field staff in Guatemala noted that the MUAC Z-score measurements which were discordant with BMI Z-scores occurred at the borders of two colors. This misclassification of some of the MAM and SAM cases indicates that a protocol to classify borderline cases may be required to enhance concordance between the measures. Other possible solutions include repeating measurements, or measuring the opposite arm as well and recording the more extreme of the two. Also noted, the nature of discordance did depend on nutritional status to some extent. MUAC Z-score was more likely to categorize an undernourished child into a more severe classification and an overnourished child into a less severe classification, a finding that coincides with previous studies [33]. Recommendations from the Guatemala field staff to improve the training and ultimately the effectiveness of the MUAC Z-score tape in a community setting include offering a reference pamphlet to the volunteers, which shows the exact version of the MUAC Z-score tape are using, with both sides and corresponding colors. They also noted that the practice sessions were very helpful and one of the most important aspects of the training. Having a doctor, nutritionist, or someone with medical experience leading the process was important during the training. Another benefit of the trainings noted by the field staff was that empowering volunteers and community leaders who have no previous formal medical training foments trust that may improve the quality and efficiency of community projects.

Of note in this study was the high prevalence of “at risk” of overweight and overweight. In contrast to other countries in Latin America where overweight and obesity are rapidly increasing, Guatemala has one of the lowest prevalences of overweight, and one of the lowest prevalences of inactive people over the age of 15 [39]. However, other studies have pointed to the nutrition transition that is occurring in Guatemala toward processed (e.g., canned foods, cheeses, refined sugar) and highly processed foods (e.g., chips, soft drinks, sweetened breakfast cereals) is associated with increases in BMI [40, 41]. Because Guatemala has such a high rate of childhood stunting, the risk of the double burden of malnutrition (overweight or obesity coexisting with undernutrition) is alarming [42]. The MUAC Z-score tape used in this study not only identifies underweight, but the entire spectrum of nutritional status. The use of this specific tape has indicated that there may be a growing concern of overweight and obesity in the population of children and youth served in Guatemala, which may have implications for the field medical staff and nutrition program.

There are several limitations of the current study. One limitation is that the community volunteers were asked to measure children and youth in their communities, and were not randomly assigned participants. This may have introduced selection bias, especially if children were related or lived in close proximity, or if those children or youth who were not included were fundamentally different in ways that were related to the outcome of agreement. Additionally, there were 71 children or youth who did not complete the follow-up measurements, which could have skewed the results. Another limitation is the window of time between MUAC Z-score tape measurement and height and weight collection. Weight is affected by diet, activity level, and illness. The extent to which these environmental factors may have changed a child’s weight and thus BMI over the course of 15 days were not recorded in this study; however, their impact is likely low in this narrow timeframe. When considering the limitations of the generalized linear mixed model, we had far fewer observations in the extreme tails of the distribution than near the center, making those estimates less certain. Interpretation near the ends of or beyond the observed range of BMI Z-scores should be done with caution. We also did not have a determination of nutritional status as determined by a medical professional; we compared only anthropometric measurements. Therefore, knowing the accuracy or performance of each method is not known, but as screening methods they yield similar results. Lastly, the MUAC Z-score tape collates MUAC Z-score data delineated by age in months into ½ year or full year increments. Practical implications limit the real estate available on the MUAC Z-score tape, thus the age categories are not as granular; however, the tapes can be customized to expand or contract age ranges as was done for Children International.

## Conclusions

To the best of our knowledge, this is the first study to assess the effectiveness of the MUAC Z-score tape in a community setting using non-medical volunteers. Volunteer-implemented nutritional risk screening with the MUAC Z-score tape was effective at detecting SAM and MAM in this study.

Future directions for this work include investigating scalability of the MUAC Z-score tape to detect malnutrition in locations such as Guatemala that require cost-effective strategies to address the heavy burden. Use of MUAC Z-score tape to monitor rehabilitation progress and determine success also remains to be investigated.

## Supplementary information

**Additional file 1:** MUAC Z-Score Tape Instructions for Use. This reference document contains the instructions (English) and diagrams of portions of the MUAC Z-score tape to facilitate training and interpretation. (PDF 1726 kb)

## Data Availability

The datasets used and/or analyzed during the current study are available from the corresponding author on reasonable request. Information regarding the MUAC Z-score tape and instructions for use are also available upon request.

## Abbreviations

BMI: Body Mass Index
CI: Children International
MAM: Moderate Acute Malnutrition
MUAC: Mid-Upper Arm Circumference
SAM: Severe Acute Malnutrition
WH: Weight for Height
WHO: World Health Organization
