# Supplementary material for: Assessing effectiveness of a novel mid-upper arm circumference Z-score tape in a community setting in Guatemala
Source: Arch Public Health. 2019 Oct 4;77:44. doi: 10.1186/s13690-019-0370-0 (PMC6777036; doi:10.1186/s13690-019-0370-0)
Supplement: Supplementary file 1 — Additional file 1: MUAC Z-Score Tape Instructions for Use. This reference document contains the instructions (English) and diagrams of portions of the MUAC Z-score tape to facilitate training and interpretation. (PDF 1726 kb) [file 13690_2019_370_MOESM1_ESM.pdf]

MUAC Z-Score Tape

Instructions for Use

1. Your MUAC z-score tapes may arrive individually or in sets containing 1 2-sided infant device and 1 2-sided child device. If it comes as a set you will want to separate the devices.

2. Familiarize yourself with the key that orients you to the age group, the colors and the markings. Note that the 2 green arrow heads represent the zero-end of the tape measure and the the place where you read the patients MUAC and z-score. To measure, slide the tail end of the tape into slit "A" and back out through slit "B" to create a loop.

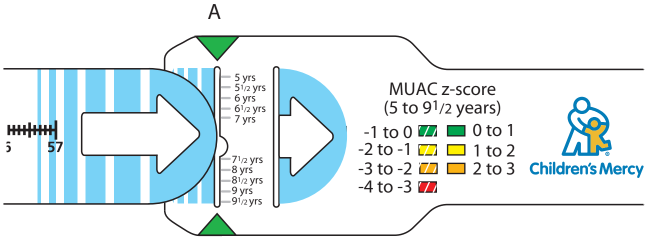

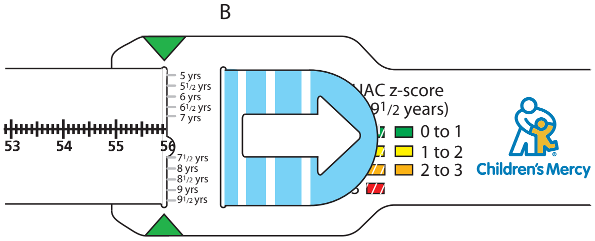

3. Identify the midpoint of the upper arm between the acromion and the olecranon process (the ruler on the device can be used to assist with this process) and slide the loop of the device up to the midpoint. You must make sure that the elbow is fully extended (in other words that the arm is straight for before measuring). Pull the tail end of the tape until it is snug but does not compress the skin.

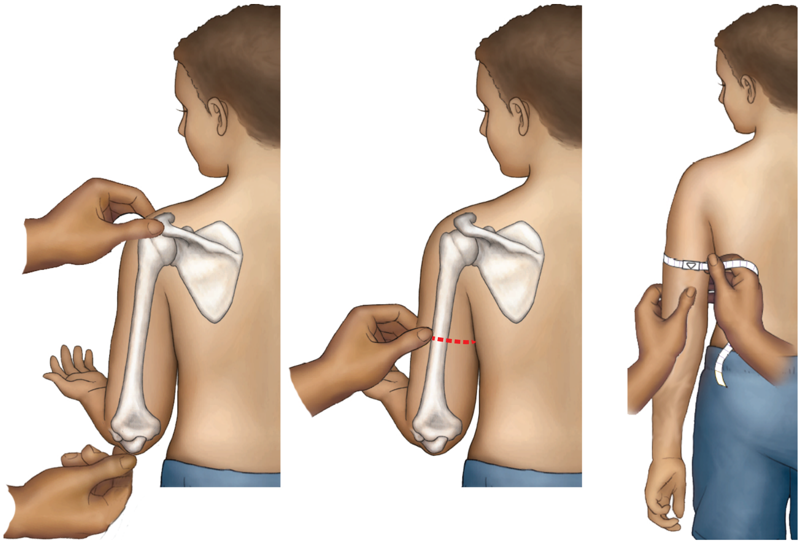

4. Identify the color band corresponding to the age of the patient. In the example below the MUAC is 16.6 cm. The MUAC z-score ranges and the classification for children of varying age are provided in the adjacent table.

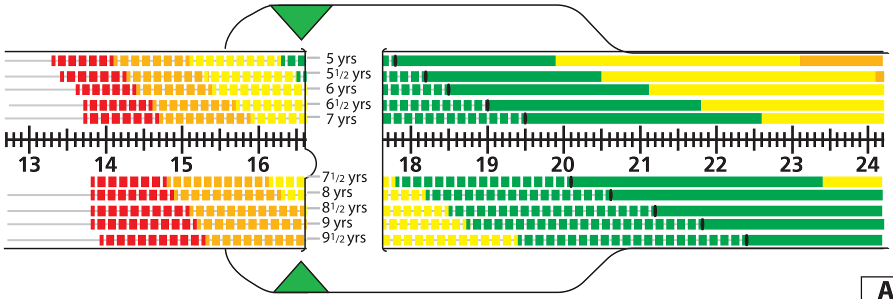

Age (yr)	MUAC z-score range	Classification
5	0 to -1	normal
7	-1 to -2	mild malnutrition
9	-2 to -3	moderate malnutrition
